# Tales from three countries: reflections during COVID-19 for mathematical education in the future

**DOI:** 10.1007/s10649-021-10066-9

**Published:** 2021-06-09

**Authors:** Christina M. Krause, Pietro Di Martino, Judit N. Moschkovich

**Affiliations:** 1 University of Duisburg-Essen, Essen, Germany; 2 University of California, Berkeley, USA; 3 Università degli Studi di Pisa, Pisa, Italy; 4 University of California, Santa Cruz, USA

**Keywords:** Goals of school mathematics, Active citizenship, Crisis, Mathematical literacy, COVID-19

## Abstract

How can school mathematics prepare citizens for a democratic society? Answers to this question are not static; they change as society and its problems change. The SARS-CoV-2 pandemic with its corresponding disease COVID-19 presents such a problem: what is needed to navigate this complex situation that involves, among other things, mathematics? Using the essay genre, we use three narratives from three countries—Italy, the USA (California), and Germany—to reflect on the goals of teaching mathematics during this crisis and examine aspects of each country’s standards for mathematics education. These three stories are framed by the authors’ backgrounds, experiences, interests, their country’s situation, and response to the pandemic. We first present the three narratives and then examine common issues across them that might provide insights beyond this current crisis, for preparing students to become active citizens. In particular, we focus on three issues: (1) developing a positive mindset toward mathematics to engage with and reflect on real-world problems, (2) improving interdisciplinary connections to the sciences to better understand how science professional practices and insights are similar or different from everyday practices, and (3) considering interpersonal and collective matters beyond the individual.

*“Epidemics, like disasters, have a way of revealing underlying truths about the societies they impact.” — Anne Applebaum, The Atlantic, March 2,* 2020

The SARS-CoV-2 pandemic has had a massive impact on social life, economics, politics, and education. This dramatic event brought us to reflect on issues with new urgency, using our own (sometimes very personal) experiences of such extreme times. Beyond those of immediate interest and relevance—the *how* of teaching as challenged in distance learning—the *what* of learning becomes an important issue in a long-term perspective. This essay re-visits the role of mathematics education in preparing students for active citizenship in the particular context of these peculiar times.

As educators, we ought to contribute to building a society of individuals who can reflect upon information, solve problems, or develop other higher-level critical thinking skills (Paul & Wang, 2006). We build on previous work in mathematics education that calls for schooling to contribute to developing constructive, engaged, and reflective citizenship, giving individuals the tools to recognize, apply, and interpret mathematics in a variety of contexts in the everyday world (Niss & Jablonka, 2014; OECD, 2013, 2020) to make informed decisions in their everyday life. Many scholars have elaborated on this sentiment over recent decades. Niss (1996) underlines that one main goal for teaching mathematics to all must be to provide individuals with the essential knowledge, skills, and competencies to exercise an active citizenship in a variety of situations . The idea of “mathematical literacy” as considered by Niss and Jablonka (2014) focuses on mathematics as a tool for effectively navigating real-world situations and problems. They acknowledge “the importance of flexibility in putting mathematics to use in novel intra- and extra-mathematical contexts and situations” (Niss & Jablonka, 2014, p. 393) for mathematics education. However, mathematical literacy is a dynamic concept which needs to be re-examined, updated, and adapted to particular settings (Jablonka, 2003) and to new challenges (Kilpatrick, 2001). Niss and Jablonka (2014) furthermore highlight that “identifying the demands and knowledge bases for mathematically literate behavior in different contexts remains a major research agenda” (p. 395). New scenarios, like this pandemic, force us to reflect on the goals of school mathematics and how they relate to supporting participation in a modern democracy.

## The challenge of the current crisis

All over the world, restrictive measures have been taken to contain further spread of the virus. In democratic societies, we cannot expect citizens to accept and follow such measures uncritically, without a basic understanding of the situation and being able to follow the discourse around the measures to not fall victim to fear mongering or “fake news.” Mathematics plays a crucial part in this public and personal discourse, in describing and modeling present and potential future scenarios to explain and justify regulations and potential restrictions imposed on society. No mathematical task we can create could be a richer application of mathematics than this real situation. At the same time, the consequences of not being able to appropriately navigate this mathematics can be severe. Being a mathematically literate citizen is crucial for understanding current phenomena and decisions, for distinguishing opinion from fact-based argumentation, for making day-to-day decisions, for anticipating the consequences of and outcomes from those decisions, and for planning the future on the basis of different possible scenarios.

As mathematics educators, we have felt challenged and frustrated during this pandemic, experiencing and noticing a lack of mathematical (and scientific) literacy that seemingly stands in the way of effectively facing a global crisis. As researchers, observing this absence, we landed on the question “(How) can we do better?” as a way to contribute to our communities’ collective reflection.

We address this question in our multi-voiced essay from three national perspectives based on personal observations of public and personal discourse within three countries—Italy, the USA (California), and Germany—narrated and reflected on by each of us as we were experiencing the crisis. In each country, the story of the pandemic and of how people were dealing with it has been different, and so are the three narratives capturing each author’s experiences during the pandemic’s very early stage.^1^ “The aims of mathematics teaching cannot be meaningfully considered in isolation from their social context” (Ernest, 2000, p. 7), and the goals of school mathematics are always influenced by cultural and social values of the respective society (Niss & Jablonka, 2014). Our individual reflections tie the narratives to the standards for school mathematics in each country and consider how those standards can contribute to preparing active citizens to responsibly navigate the pandemic. With this, we address what the call for this special issue describes as “critical reflection on curriculum and how it prepares or fails to prepare society for this and other global crises.”

We believe that the style of this piece, a reflective essay grounded on three personal narratives, is appropriate for interrogating the events as it grounds our reflections in ethnographic details and methodologies of narrative research, and allows us to both feature and merge our voices (Barwell & Reid, 2019). We developed this paper in two steps. First, each of us wrote the personal narrative—including a brief description of the national scenario of the pandemic and an outline of how each of the national standards for school mathematics approaches the preparation of students as citizens. We then shared our narratives and reflected on what we saw as common that echoed across the three narratives. The structure of this paper reflects this process in that we first present the three individual narratives, not only from a different setting but also each written in a different style. We follow those with a discussion of how our narratives and reflections might inform mathematics education for citizens of a global democratic society facing problems beyond the immediate crisis.

## Three countries, three voices, three narratives

### An Italian story (P. Di Martino)

In Italy, we suddenly passed from a state of relative tranquility to a state of uncertainty and fear. In this scenario, the first reaction was touching: the voice of the Italians singing from their balconies across the country resonating in the empty streets will remain in the memory of many people. In this scenario, the unity of intent of the entire country is essential: however, as time passes, the emotional impact is no longer sufficient to guarantee this unity. It is crucial that everyone has the competence needed to understand the situation and act appropriately. It is exactly in this context that I had the opportunity to reflect on the role mathematics takes in navigating this complex scenario of the pandemic, how this role can be identified in the public discourse around managing this situation—and the critical issues it reveals with respect to aspects of mathematical literacy as addressed in the Italian curriculum.

#### The Italian context—the pandemic scenario

Italy was the first Western country to suffer a coronavirus emergency and adopt severe measures to contain the spread of the virus. On March 4, 2020, in a dramatic television appearance, the Italian prime minister announced an unprecedented measure in Italian history: the lockdown and the immediate closure of all schools and universities. Considering the economic impact, and also the encouraging quantitative data about the increased number of recovered cases and the decrease in hospitalized people, the Italian government announced the beginning of the long-awaited “Phase 2” for May 4, 2020, including the reopening of a majority of businesses.

After nearly 2 months of strict lockdown, Italy reported more than 200,000 confirmed infections with the new coronavirus, more than 30,000 deaths related to COVID-19, and more than 100,000 people still recovering in May 2020. While writing the first draft of this paper, Italy and Italians have just entered Phase 2 of the lockdown with a mix of hope and fear. The numbers related to the virus still continue to be the protagonists as the schools are supposed to re-open after the summer break in September 2020.

#### The Italian context—the national standards

The Italian standards for the first (grades 1 to 8) and second cycle (grades 9 to 13) of education (INDIRE, 2014) capture the goals of school mathematics in the sense of mathematical competence as defined in the EU document “Proposal for a Recommendation of the European Parliament and of the Council on key competences for lifelong learning” (Education Council EU, 2006):

Mathematical competence is the ability to develop and apply mathematical thinking in order to solve a range of problems in everyday situations. Building on a sound mastery of numeracy, the emphasis is on process and activity, as well as knowledge. Mathematical competence involves, to different degrees, the ability and willingness to use mathematical modes of thought (logical and spatial thinking) and presentation (formulas, models, constructs, graphs, charts). (p. 15)

I believe that the pandemic has shown how different an “everyday situation” and the problems emerging in it can look like and that it, thereby, has given another perspective on the competencies needed. Its public discourse in Italy and issues in the relationship between information and personal responsibility required to make decisions based on mathematics provide an occasion to concretize the rather broad mathematical competencies and identify some gaps in what is needed. From that, we can reflect on the potential and needs of mathematical literacy as developed through school mathematics in Italy—in this case “to develop and apply mathematical thinking in order to [contribute to solving this problem] in everyday situations.”

In my observations, I identified three main issues related to the mathematical competence of the people related to three actions: understanding, questioning, and steering a complex situation.

##### Issue 1: mathematical competence to understand the phenomenon at a basic level.

Mathematics dominated media communication about the new coronavirus: for almost 2 months, the days in Italy were marked by the Civil Protection bulletin on TV at 6 p.m., which informed Italians about the numbers of this tragedy. Virologists and other experts—constantly invited in popular TV shows—used numbers and mathematics to explain the need for the lockdown measures. They mentioned the basic reproduction number (*R*_0_)—the average number of infected per positive individual—discussing the maximum of a (hopefully limited) function that describes the spread of the contagion. Although all of the necessary mathematics to understand these aspects is included in the Italian standards, reading the newspapers and posts on social networks revealed that a majority of the Italian people had difficulties comprehending the mathematical concepts involved.

Is this lack of knowledge among the adult population worrisome? Surely it is; however, I argue that this dramatic experience has shown that the lack of specific knowledge is not the main issue in people’s mathematical literacy.

Let us consider a specific example: the comprehension of the spread of the contagion was and is crucial in order to *participate in solving the current problem*. In this case, it appears sufficient to comprehend that:

1. The model for the contagion initially is exponential (depending on the *R*_0_value) and an exponential function grows very fast;
2. The parameter *R*_0_ *evolves* in the parameter *R*_*t*_ (the average number of people who become infected by an infectious person at the specific time t). The value of *R*_*t*_ is strongly affected by people’s preventory habits, including social distancing and wearing a mask, leading to *R*_*t*_ becoming smaller.
3. Small variations of *R*_*t*_ have a great impact on the virus spread.

This kind of general knowledge can be easily recovered or introduced when it is useful, also supported by using graphs and images to clarify. It is not necessary (nor possible) an a priori and generalized *omniscience*, but rather the willingness in recovering mathematical arguments when needed: this is an affective issue of mathematics education more than a cognitive one. While this is included in the Italian standard for the first cycle as “the student will develop a positive attitude towards mathematics” (MIUR, 2012, p. 40), this period confirmed that this goal is far from being achieved: the wide spread of a negative attitude toward mathematics keeps many people away from any mathematical—and more in general scientific—argument (Di Martino & Zan, 2010), eventually preventing a solution of the problem on a societal level.

##### Issue 2: mathematical competence to question phenomena.

**Fig. 1:**
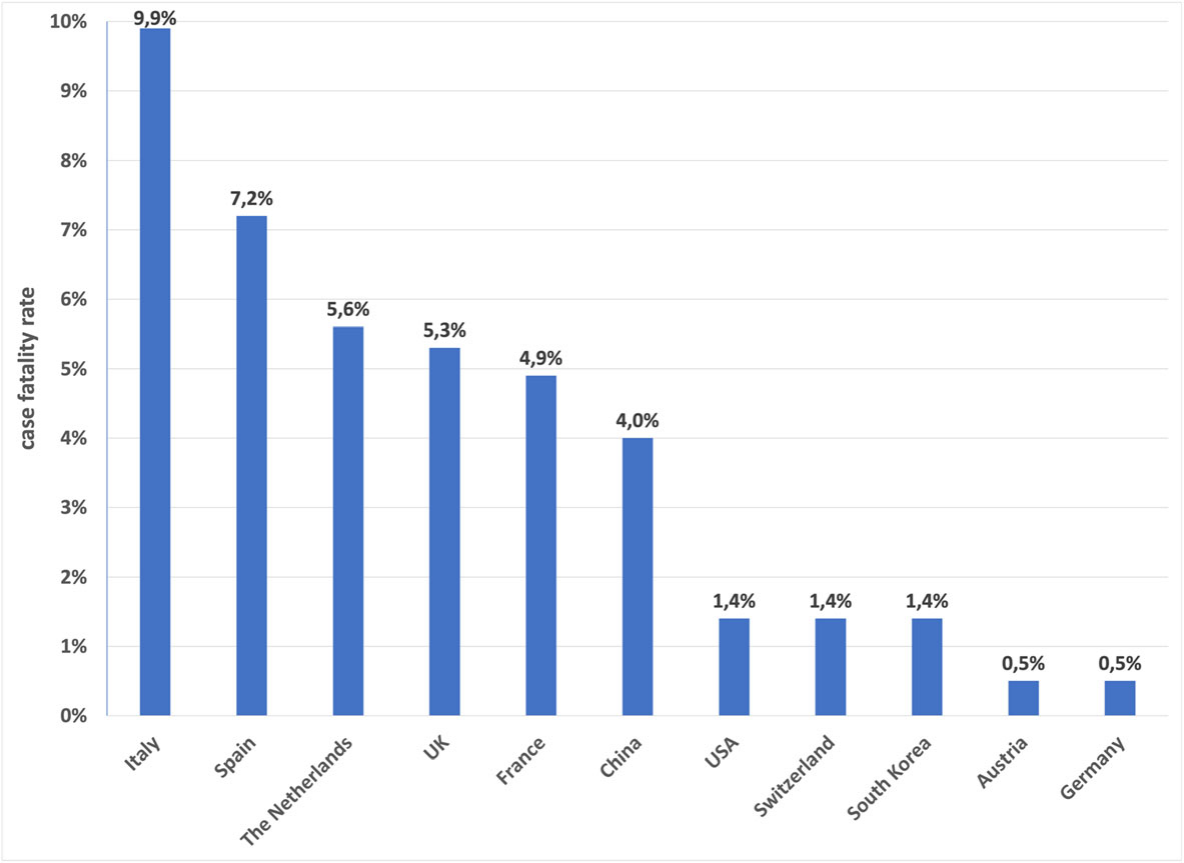
COVID-19: case fatality rate in some countries (following Villa, 2020)

The mathematical competence of interpreting and questioning graphs appears to be particularly relevant during this pandemic for practicing active citizenship and understanding the narration of the phenomenon. On March 27—20 days after the lockdown announcement in Italy—some media published the histogram proposed by the Italian Institute for International Political Studies (IPS, see Fig. 1).

This graphical representation highlighted massive differences between data from the involved countries: For example, it seems particularly remarkable that Italy appears to have a case fatality rate 20 times as high as Germany, despite the countries’ geographical neighborhood! It is quite obvious that the perceptual impression only tells a part of the story or, moreso, that we need to interpret the reasons for this discrepancy.

In this case, the case fatality rate denotes the proportion of deaths from a disease compared to the total number of confirmed positive cases (being different from mortality rate, which compares to total population). The mathematical competence now allows to interpret the differences in the graphical representation in Fig. 1 on two levels: first, the differences as they might be related to contextual aspects, like different average age, or differences in the healthcare systems; second, considering differences in the ways the countries collect the data (the number of tests performed was extremely variable and this affects the denominator). These two different levels are not mutually exclusive. However, to better interpret the graph, they both need to be explored.

The interpretation of these kinds of graphs puts in perspective “the ability and willingness to use mathematical modes of thought [...] and presentation” (p. 15) mentioned in the EU Education Council document and the primary (mathematical) need to clarify what the representation talks about.

##### Issue 3: mathematical competence to make appropriate decisions in problematic situations.

While the focus in Phase 1 was to use mathematics to explain facts and phenomena related to the new virus and its spread, this focus shifted when approaching the second period (in the second half of April 2020): now, mathematics became crucial for imagining and planning how to gradually open up the country again. In this phase, the mathematical competencies of decision makers of a nation become of crucial significance, as for them, making the right decision and communicating them to the people efficiently requires a more comprehensive understanding of the complexity of an intricate situation than can be expected from the general public. In particular, a lapse or a non-scientific approach adopted by them due to their own lack of understanding can have serious consequences, as illustrated by the following two episodes of politicians’ faulty use of mathematics.

1. During a live TV address, the welfare councilor of Lombardy tried to appease people, saying that since the value of *R*_*t*_ was 0.51, it takes two infected people to infect a non-infected person, but that fortunately, it is particularly difficult to meet two infected people simultaneously. As it appears evident, this is not just a gross mathematical error (*R*_*t*_ is a mean value and, obviously, it is not additive): such a misuse of mathematics is particularly concerning considering that people might rely on his word, potentially leading to behavior that could endanger a region already severely hit by the virus.
2. The Italian Minister of regional affairs told the press: “In view of Phase 2, I ask the scientific community, without controversy, to give us irrefutable certainties and not three or four options for each topic.” The Minister would pretend to have elements for making decisions under a condition of certainty. Unfortunately, decisions are rarely made under certainty in real life when the problems to be faced are complex. In my view, mathematical competence should develop on the one hand the awareness of the limits of modeling: no model can represent reality in its complexity; on the other hand the competencies for decision-making under risk or even indecision. While school mathematics almost always focuses on certainty, even within the study of probability, we might need to reflect much more on uncertainty and on the limits of modeling. In fact, one of the competency development goals for mathematics at the end of the lower secondary school in Italy is supposed to address exactly that: “in situations of uncertainty (everyday life situations, games, etc.) he/she is oriented with probability assessments” (MIUR, 2012, p. 41).

#### Reflecting on these issues to look to the future

From the observations I described and my reflections on them, two main points have emerged for me during this pandemic and related to the Italian context—the first one concerning attitude toward mathematics, the second mathematical literacy of decision makers.

- People’s reactions in this pandemic underlined the spread of a widely negative attitude toward mathematics among the adult population in Italy. We have witnessed the proliferation of strange, unscientific, and dangerous theories but also the risk of refusing to approach facts that involve math and the resulting dependence to fully rely on others when mathematics is used to justify decisions. Also, on this occasion many people showed their own fear of math and their rejection of mathematical arguments as relevant factors in justification.
- The mathematical competence of those decision makers. The described episodes (and others similar) are particularly worrisome and recalleded the words Vinner pronounced during its plenary at ICME 9 (Vinner, 2000) about the mean mathematical competence of politicians. The pandemic not only sadly confirmed the picture drawn by Vinner; it also showed the importance of having authorities equipped with a good mathematical competence (or with a trained staff).

These two points interact at large: A negative attitude makes one reluctant to engage in the mathematical discourse and argumentation, leading to rejection of arguments and measures that are based on mathematics (Di Martino & Zan, 2010). Then, again, the lack of mathematical competence displayed by decision makers on the one hand highlights the risk of “relying on” others without control; on the other hand, it potentially weakens people’s trust in mathematics even more.

Observing the widely shared negative attitude shows that the following goals in the Italian curriculum for the first cycle are still wishful thinking: “Gradually, stimulated by the teacher’s guidance and the discussion with peers, the student will learn to deal with problematic situations with confidence and determination.” (MIUR, 2012, p. 39). My reflections however emphasize the strong interaction between willingness and abilities mentioned by the Education Council EU (2006) and the need for further emphasis on especially the former in this pair, to not have a lack of attitude stand in the way of responsible decision making on large and small scale.

### Stories from the USA (J. Moschkovich)

This section is a set of ethnographic memos written during the first few months of the pandemic. The memos raise more questions than they answer and are ordered chronologically to show how my own questions changed over time. The memos are then followed by my reflections on what connections, or lack of connections, I saw between the US mathematics standards and the questions I raise in the memos.

#### The US context—memos from early stages of the pandemic

The USA is a country with great variation in population density, resources, and political leadership. The first clusters and “hot spots” were reported in early March (Seattle, WA and Santa Clara County in California’s Silicon Valley). The US response to the pandemic started comparably late, although California led the way with the earliest calls to “flatten the curve”^2^ and orders to “Shelter in Place”^3^ on March 19.^4^ I include some personal background, to contextualize my memos. I am originally from Argentina, so I watch the pandemic situation in Argentina and talk to my extended family there. I was born in 1956, shortly after the last polio epidemic in Buenos Aires, placing me very close to the “65 and over” vulnerable group. I currently live in a politically progressive smallish town in California, close to Santa Clara county, and that is where I wrote the following memos.

##### Memo 1: Why am I constantly looking at graphs?

 March 5, 2020: No “Shelter in place” order yet, but I am strongly wishing there was one. Just returned from a trip to Vancouver and I am watching the models, *all* the models I can find, but especially those that I think I can trust. At first I think “Graphs make me calm,” then I realize that they actually do not. Graphs do not reduce the uncertainty for me, but they do help me imagine the future, a future that includes not only the bad outcomes but also the good outcomes, if humans act in sensible ways. Then along comes the “flatten the curve” refrain. I wonder, what do others see in that curve? I imagine the collective; I imagine what happens to others who are not me. Do others see the collective in a graph of the data? People mention that the curve is growing exponentially. I wonder, who really understands the difference between linear versus exponential growth? The difference is not intuitive; if it were, everyone would pay their credit card balance each month. I would guess that lessons in high-school consumer math classes have tried to address this issue, but do any lessons apply to public health issues or collective problems such as an epidemic?

##### Memo 2: This is not just math; it is science and a very human science at that.

March 16, 2020: Day 1 of “Shelter in Place” in California. Numbers only reflect what we can count: i.e., modeling is based on imperfect data (no tests available, deaths that are not recorded as due to COVID, etc.). Medical science is observational and depends on what questions we ask: Who is dying? What ages? What gender? What co-morbidity factors? What zip codes in New York City? Which social classes? Which ethnic groups? Which locations? Where are the largest hot spots? Cruise ships, prisons, nursing homes, and meat packing plants. What questions do I need to ask to understand *why* these are hot spots? Although the Common Core State Standards (CCSSM; National Governors Association Center for Best Practices & Council of Chief State School Officers, 2010) math practices^5^ say nothing about asking good questions, the Next Generation Science Standards (NGSS Lead States, 2013), on the other hand, start with “Asking Questions.” I read a piece in the New York Times about people wondering why the total number of cases can be so different in neighboring counties. In which class in secondary school (biology or social studies) would I have learned that it is people, not the virus, that cross county lines?

##### Memo 3: Which numbers do we watch?

Date is unclear, every day feels the same, and many (including me) are making calendar mistakes (i.e., showing up at a zoom meeting on the wrong day or time). A friend’s daughter is an emergency room physician in a large city on the East Coast. She tells me she is worried because the number of total cases is rising. My nephew is comparing three locations to decide where to shelter and we look at the numbers together. I say that for me this is about proportional reasoning. Watching total numbers is misleading: density matters, the number of reported cases per 100,000 people matters, the percentage of deaths per 100,000 people matters. Also how fast death rates are increasing matters. All of these are complex ideas, not easy to understand, keep in mind, or compare. Logarithmic and linear graph scales are not easy to move between. Do other people even notice when the scale is linear and when it is logarithmic? I imagine there must be research on that, but at this point in the pandemic, I do not have the energy to look up all the research I wish I could cite that relates to a lack of understanding the mathematics.

##### Memo 4: What do I know about statistical models that I wish everyone else did?

 April 22, 2020. The New York Times shows 5 different models. Which one do I trust the most? Models are based on incomplete data. What are my own or others’ beliefs about science? Overall, science changes as we collect more data, so we cannot expect the same answer each day, which bothers many lay people who wish that science would just give us one definite answer.

##### Memo 5: Intuition and statistics are not good friends (or the importance of testing, sampling, and countering intuitions).

May 18, 2020. California is starting to relax “Shelter In Place,” and it is left up to local authorities to make decisions regarding how to “open up.” Out for a walk, I run into an acquaintance; she is with a friend from out of town. My friend says “This is my friend from another town in California.” I ask “How is your town?” They answer “There are no cases where I live.” I politely say, “There are no *reported* or *confirmed* cases, the testing rate in California is less than 1% so there may be cases, but without more testing we cannot know for sure.” On another walk, a neighbor says “The virus is not in our town.” I explain to my friends and family why opening up without sufficient and reliable testing is not scientifically sound. I watch a webinar by a local biologist who is an expert and cry when I hear the clear explanations and see the amazing graphs. He reminds us that any model needs to specify what the conditions are, i.e., testing, how much, sheltering, what kind, etc. I conclude that intuition and statistics are not good friends (even for mathematically educated people). I have read Kahneman’s book *Thinking, Fast and Slow* (2011). I know about the base rate fallacy, the law of small numbers, and other typical counterintuitive statistics pitfalls. However, I do not have a good simple example that I can use to explain to my friends or family why it is important that the “positivity” rate in California be closer to 10% than the current 20%.

#### The US context—the national standards

The mathematics education policies in place in the USA at the time of the pandemic include the Common Core, especially the eight Common Core State Standards (CCSSM; National Governors Association Center for Best Practices & Council of Chief State School Officers, 2010) Mathematical Practices. Research in mathematics education has proposed that mathematics instruction in schools should reflect the practices of mathematicians (e.g., Cobb et al., 1993; Lampert, 1986, 1990; Schoenfeld, 1992) and emphasize classroom activities that are developmentally appropriate approximations of disciplinary mathematical practices. The Common Core Standards for Mathematical Practice have important precursors, for example, in the NCTM Process Standards (NCTM, 1989, 2000) and habits of mind (e.g., Driscoll, 1999), suggesting students participate in practices such as conjecturing, constructing arguments, and revising arguments in a classroom community (Moschkovich, 2013; Schoenfeld, 1992). These mathematical practices provide learners with access to approximations of the powerful habits of mind underlying disciplinary practices. 

The eight Common Core mathematical practices are complementary to the five mathematical proficiency strands (Kilpatrick et al., 2001). The strands focus on the cognitive and the individual, using words like ‘reasoning’, ‘fluency’, and ‘understanding’. The practices listed in the Common Core can be interpreted in multiple ways, depending on how they are (or are not) theoretically framed. From the Vygotskian perspective I use in my work, mathematical practices (including those listed in the Common Core) are sociocultural and collective practices (Moschkovich, 2013, 2015). From a Vygotskian perspective, mathematical practices are socio-cultural phenomena in that they are higher order intellectual activities and originate in social interaction. They are first constructed interpersonally and then appropriated to become part of the repertoire of practices that an individual will use. A Vygotskian theoretical framing clarifies the concept of mathematical practices. First, it provides a practice perspective on cultural activity. Second, it provides a connection to discourse as a central aspect of practices (Moschkovich, 2015). And lastly, it describes how practices are acquired through appropriation (Moschkovich, 2013). I have always seen these mathematical practices as foundational, not only for participation in STEM disciplines, but perhaps most importantly, for participation in a democracy. During this public health crisis, I saw some “thinking practices” in my memos which are broader and different than any mathematical practices. This insight informs my response to the question “What math education do future students need?”

#### Reflections for future mathematics education

First, separating mathematics from its applications is foolish, either during a pandemic or in educating students to participate in making decisions in a democracy. Science literacy matters, and not only natural science (biology) but also social sciences: epidemiology requires mathematics, biology, and social science together. Second, we need to learn what questions to ask and what data to collect, not just how to interpret graphs or what answers to trust. Third, we need to learn to perceive with open minds so that our intuitions both guide us and teach us what to question. Lastly and perhaps most importantly: we need to develop compassion. Compassion for ourselves: we cannot possibly understand a pandemic all at once. Compassion for science and scientists: it and they have their limits. And compassion for others who are not like me: so we can think well about the collective (and not only the individual). What course, in what school, in what country, is currently addressing competency in compassion? When I teach the next group of pre-service secondary mathematics teachers, I will be asking them that question.

### A German story (C. Krause)

In the process of writing the first version of this manuscript, I relocated from the USA to Germany. While living and working in California, I closely followed the situation in Italy and Germany—having lived in both countries and being connected to them through work, past life, friends, and family. Germany seems to be prepared rather well. Being warned by what happened in their European neighborhood, they acted early. I am happy to read about the relatively “good” numbers—my home country seems to give a good example of how to manage the crisis. However, I have a hard time fathoming the reports I see about the resistance against simple (and comparably light) measures implemented in Germany, like staying at home, staying apart, and wearing masks. I could not help but wonder how mathematical education matters in this current situation.

#### The German context—a scenario of the pandemic

In late May, Germany reported about 181,500 confirmed positive cases of SARS-CoV-2, 8500 related deaths, and estimated 162,800 active cases. With the absolute number of confirmed cases not necessarily considered small, comparison with death toll showed a low case mortality rate in relation to other countries. At the same time, Germany might have a lower number of undetected cases, as they conducted a comparatively high number of tests (per 1M population) already relatively early on.

Different measures were put in place on a federal level to achieve the goal of reducing the spread of the virus. In February/March—around the time when Italy went under lockdown—people were first advised to follow strict hygiene standards, to work from home, and to distance physically (“social distancing”) as much as possible. Reacting to the evolving situation in other countries, schools, childcare facilities, and businesses were closed (mid-March) and restrictions to public and personal gatherings were announced.

As of June 2020, the federal states are slowly opening up, with schools teaching in shifts and non-essential businesses operating again. Policies for wearing facial masks are in place. The current situation can be described as good in comparison to other countries. However, the situation is fragile with people getting impatient, pressing for a quick reopening without measures, and thus potentially risking a second wave of infections that experts warn could potentially be more severe than the first one.

#### The German context—on a peculiarity of the national standards

German goals for school mathematics are deeply influenced by the idea of “Allgemeinbildung.”^6^ This idea became historically relevant in the German tradition of mathematics education (see Biehler, 2019; Jahnke, 2019 for the historical development and background, Biehler, 2019 for the relation to mathematical literacy) and therefore became a cornerstone in all three documents of the Bildungsstandards after primary education^7^ (KMK, 2003, 2004, 2012). While there is no suitable English counterpart to the concept of Allgemeinbildung (see Niss in Biehler (2019)), it is defined by Winter as “knowledge, competencies, and skills that are essential to every human being as an individual and as a member of societies independent of their gender, religion, and profession, etc.” (Winter, 1995, p. 37, translation by CK). With contributing to establishing Allgemeinbildung one of the educational goals in Germany, mathematics teaching should always justify how it contributes to an autonomously thinking—an enlightened—citizen:

Can the teaching of mathematics [...] help to develop the faculty of judgement in matters of public life? In short: can it contribute to *enlightenment*? (Winter, 1990, p. 131, in Biehler, 2019, p. 149)^8^

“Enlightenment,” writes Winter, “is civil right and civil duty (and is indeed not thrown into one’s lap).” (Winter, 1995, p. 38, translation by CK) It is the basic requirement for a functioning democracy:

Democracy can only be imagined as a society of responsible persons. Winter considers the participation of citizens in public affairs under the perspective of *the tension between experts and non-specialists*. Most political decisions require highly specialised knowledge which is provided by experts, whereas in principle all members of society are supposed to decide upon political and social matters and at the same time are laymen regarding most questions. Consequently, in a democratic society, non-specialists should be qualified to understand how experts arrive at their specialised knowledge, how safe this knowledge is and to ask critical questions, in short: *citizens should develop a faculty of judgement*. (Biehler, p. 149, emphases added)

In the current situation, this ‘faculty of judgement’ (in the sense of Winter and Biehler) is reflected in the ability to act based on decisions informed by critically evaluating one’s own and others’ solutions, and the contexts in which models are built and interpreted. Despite the high transparency of the scientific discourse and the increased commitment and effort to making people understand why certain measures are taken, the tension between experts and non-specialists is observable in a strong resistance against these measures. People question the severity of the disease to be high enough to justify the measures, claim it is no different from influenza, push back against distancing rules and mandatory facial covers, and lately gather for protests to shut down all restrictive measures. Although the majority of people seem to comply with the temporary rules, the active pushback appears to be much higher than in other (European) countries.

Observing the situation in Germany, I wonder what ‘faculty of judgement’ is needed in the current situation and how it can be supported by mathematics teaching. While there certainly is much more to the relationship between mathematics education and Allgemeinbildung with respect to the current situation, I will focus on two points in my reflection on the relevance of a ‘faculty of judgement’ in this crisis.

##### (1) Perceiving and understanding the situation through mathematics as basic requirement for ‘faculty of judgement’.

All three documents of secondary education in Germany refer to three “basic experiences” that, following Winter (1995), school mathematics should offer rich opportunities for. The first one is to “perceive and understand the phenomena of the world around us in nature, society and culture in a specific [mathematical] way.” (p. 35, translation by CK) But what exactly can this mean in the current situation?

I happily noticed an increasing public interest in mathematics and statistics and a transparent scientific discourse and communication of the background of the pandemic. An internationally respected virologist researching the virus delivered regular podcasts, talking about the science behind the disease and how it relates to measures taken, and reported on the latest insights. Science communicators explained mathematical models of disease spread, the mathematics behind “flattening the curve” and discussed different scenarios of developments in the near future related to both. Science and math were the focus of more attention than usually, accessible in German for a wider public in real time. But can we expect everybody to follow this discourse comprehensively?

Understanding the current situation highlights a certain understanding of mathematics and statistics, such as exponential growth as rate for virus spread, percentages that denote case fatality rates, social network theories to trace virus spread,... Suddenly, everything seemed to be related to mathematics. Although much of this mathematics might not be advanced, the sheer amount of different mathematical topics can be overwhelming, with a new aspect in focus every day, and different mathematical ideas combined (e.g., exponential growth and network theory for understanding the effect of distancing measures). Furthermore, not only the mathematics in the discourse changes; the scientific insights about the virus, how it spreads, and the effectiveness of the measures develop. People, including politicians, criticize researchers for “constantly changing their opinion,” revealing a problematic attitude toward the nature of scientific results. Results in science are always preliminary, and therefore, mathematical models are always only as accurate as the scientific knowledge at the time and need to be adjusted to the circumstances.

When evaluating the situation and its communication, one also needs to keep in mind that the “tension between experts and non-specialists,” mentioned by Winter, also exists between what the general public might perceive as experts. Experts are not experts in everything. Virologists are not necessarily experts in mathematics, public health experts are not virologists. Mathematical models are based on assumptions and conditions. Simplification for easier communication might lead to comparing apples to pears without even knowing. This might cause a discrepancy in public statements, opening doors for skeptics and a mistrust of complicated models.

##### (2) Developing a mindset toward solutions for the collective for a ‘faculty of judgement’.

The idea of Allgemeinbildung in German mathematics education concerns the relationship between citizens and mathematics and the role of mathematics education in society. Developing Allgemeinbildung is meant to enable us to make informed decisions that are not only beneficial for the individual, but that also consider the society the individual is a part of. Considering Allgemeinbildung important for the goals of school mathematics also needs to uncover the collective beyond the individual. In the current situation, the actions of the individuals determine the success of the measures for the collective.

For once, this concerns the way mathematical facts are interpreted. Mathematical facts and results are often perceived as objectively drawing a clear and obvious picture. Often, this becomes expressed in references to the statistics, claiming that “numbers don’t lie.” After all, “the case fatality rate is not higher than that of the flu.” Besides many other things being concerning about this argument, this case fatality rate can be understood in multiple ways: as *one’s risk* of dying if infected, or as the *percentage of people* that will die when infected. While both are correct interpretations, the former focuses on making decisions for the individual, the latter on decisions for the collective. A society whose citizens value the collective needs to push problem solving toward situations of decisions that connect the individual and collective good, challenging what it means to “acquire problem-solving skills (heuristic skills) that go beyond mathematics, through involvement with tasks” (Winter, 1995, p. 35, own translation).

### Reflections on mathematics education’s contribution to ‘faculty of judgement’ in this time of crisis (and beyond)

Allgemeinbildung and its role in mathematics teaching highlights the need to enable citizens to develop the ‘faculty of judgement’ necessary to participate as an active citizen in a democracy during crises. My personal observations and reflections emphasized two main points:

Mathematics education that contributes to developing a ‘faculty of judgement’ in situations like the current crisis needs to emphasize flexible connections between diverse mathematical topics (that may not necessarily or obviously be linked) and gives more attention to the relationship between mathematics and sciences, for example considering models of situations under evolving conditions.

Mathematics teaching might include reflection on being a member of a society and their role within it. This goes beyond the social component of learning. It can be argued that the six mathematical process standards^9^ included in the German Bildungsstandards certainly concern competencies that develop in social contexts. But acting in social contexts does not necessarily mean acting as part of a collective. To truly consider the learner as a reflective citizen in a society in which not only individual skills are needed but also the consideration of one’s own responsible behavior within a collective, we might think further about the contexts in which these skills are developed by emphasizing collaboration, the “me” as part of a larger whole.

## Learning for the future

In closing, we reflect on the common themes that echoed across our three narratives:

The first theme is the need for developing a positive attitude and mindset toward mathematics during schooling. Studies show that many adults develop a fear of mathematics (e.g., Di Martino & Zan, 2010), and this is a clear obstacle to exercising active citizenship—a problematic factor in this time of crisis. Citizens need a ‘faculty of judgement’ to question and evaluate statements and decisions by authorities, experts, and decision-makers, understand the limits of expertise, and be mindful of and attentive to potentially faulty or oversimplified explanations by decision-makers. Those afraid of mathematics avoid engaging with mathematical arguments and, therefore, refuse to understand, evaluate, or consider mathematical messages for developing and applying a ‘faculty of judgement’. When relying on others, they are less likely to detect questionable sources.

Second, we see the need for better interdisciplinary connections to the sciences and science education, as mathematics and the sciences are all essential to understanding a pandemic and its issues. This probably most obviously concerns *mathematical models as they describe phenomena* in natural sciences, like biology, and social sciences. However, it also concerns *ways of working and thinking*, or practices. Science education emphasizes the importance of asking “good” questions, as scientists make and analyze observations. In mathematics education, this stance can be developed through problem solving and modeling. However, students cannot only answer questions asked by others or approach problems with a reproductive approach. In a democracy, citizens need to learn how to use mathematics to not only to answer questions, but also to raise them.

Furthermore, we see a need for explicit discussion of the *nature of insights* in mathematics and science and their relation to each other. Confusion about recommendations changing as scientific insights develop reveals a common perception of scientific insights as static, definite, and certain, when in fact, they are neither. Mathematical models might have to change conditions, and predictions made on those models might also need to change. Results gained using different models might not be comparable; recommendations and decisions must be made under uncertainty. A belief that science and mathematics always deliver unchangeable and irrevocable truths opens doors to mistrust and a perception that restrictions imposed on the public are arbitrary or, in worst cases, malicious.

A third thread is the need to integrate the collective in both asking questions and interpreting results. As Bakker and Wagner (2020) mentioned in their editorial on the pandemic:

We are reminded that the relation between the individual and the collective is rethought across cultures (e.g., China, South Korea, and Italy), yet universally, there seems to be agreement that the collective has to prevail in this global crisis. (p. 3)

This quote emphasizes that the current pandemic can only be overcome as a (national as well as global) society if individual decisions are made with the collective in mind. While this is certainly seen in the intentions of the measures taken, protests and reactions on social media show that this is not necessarily given on the individual level. Reflecting on the mathematics standards, we wonder if the consideration of the collective might fall short in the solution to problems solved in school. Solving problems of the collective with the individual in mind (or the other way around) might be emphasized more in preparing an active world citizen in a global society.

We also find that while a basic comprehension of the mathematical ideas used to describe this situation is important, we should not forget how complex the situation is and that we cannot—and probably should not—expect the average citizen to grasp every aspect of it comprehensively. Furthermore, this situation needs mathematics education to build up skills that allow for a critical evaluation of how mathematics is used beyond one’s own concrete disciplinary understanding.

We see the threads described above as important beyond this pandemic, in future, and continuing global crises, such as climate change and social injustice, resonating with Jablonka’s (2003) forms of mathematical literacy, among them “pursuing social change” and “creating environmental awareness.” The considerations described above can contribute to developing such broader forms of mathematical literacy. They can be understood as “proposals for change in mathematics pedagogy that would equip society better for this and other global crises,” addressing another matter of interest pointed out in the call for this special issue. Since we first submitted this contribution in June 2020, much more is happening globally than the pandemic, including protests against police violence targeting people of color. 2020 was a year of challenges, each of them providing the potential to evaluate and update our understanding of the tools needed to be an active citizen in a democracy. Much more reflection is needed on how mathematics education might succeed or fail to provide (world) citizens with those tools.

Our three narratives were shaped by our experiences and background and uncover only particular aspects of the role of mathematical education during a pandemic. Necessarily, these reflections leave out multiple other aspects not represented in the narratives. We are aware that this essay reflects only a limited view from three specific Western perspectives. Our goal, however, was not to be representative but to stimulate a wider discussion from further points of views on an international level. This multi-voiced essay is an attempt to start from what we have experienced ourselves, honoring our own personal narratives. We invite others to join this conversation using their own experiences, contributing their own perspectives, considering, challenging, and enriching the issues we raised here.

This is not a conclusion, since we are far from being finished, with the pandemic, or the reflecting, or the conversations started in this paper.

## Footnotes

1 The first version of the manuscript was submitted in June 2020.

2 This is a curve showing how the total number of infected people is growing and was used to connect that number to the hospital bed capacity.

3 “Shelter in Place” is the term in California for the US stay-at-home order.

4 Measures like the shelter-in-place order were made at the state (or province) level; decisions about rules for mandatory masks and school closures were made through a complex (and sometimes chaotic) combination of the state, county, and city levels.

5 Make sense of problems and persevere in solving them; Reason abstractly and quantitatively; Construct viable arguments and critique the reasoning of others; Model with mathematics; Use appropriate tools strategically; Attend to precision; Look for and make use of structure; Look for and express regularity in repeated reasoning.

6 Biehler (2019) suggests a translation - or rather characterisaion - of ‘Allgemeinbildung’ to ‘general Bildung’, in contrast with ‘vocational Bildung’ (vocational education) that concerns the preparation for specific vocations (p. 142).

7 Historically, secondary education was split up in three streams, resulting in three documents addressing the educational standards for school mathematics—two documents for the degrees in lower secondary and one for higher secondary leading to the German Abitur.

8 It should be noted that “enlightenment”—*Aufklärung—*in the sense of Winter should not only be seen in the individualistic sense but against its historic-political and its philosophical background (see Neubrand, 2015).

9 Mathematical argumentation; Solving problems mathematically; Mathematical modelling; Dealing with mathematical representation; Dealing with symbolic, formal, and technical elements of mathematics; Communicating mathematically.
